# Effect of Cyclic Heat Stress on Hypothalamic Oxygen Homeostasis and Inflammatory State in the Jungle Fowl and Three Broiler-Based Research Lines

**DOI:** 10.3389/fvets.2022.905225

**Published:** 2022-05-25

**Authors:** Giorgio Brugaletta, Elizabeth Greene, Alison Ramser, Craig W. Maynard, Travis W. Tabler, Federico Sirri, Nicholas B. Anthony, Sara Orlowski, Sami Dridi

**Affiliations:** ^1^Department of Poultry Science, Center of Excellence for Poultry Science, University of Arkansas, Fayetteville, AR, United States; ^2^Department of Agricultural and Food Sciences, Alma Mater Studiorum – University of Bologna, Bologna, Italy

**Keywords:** broiler chickens, jungle fowl, heat stress, hypothalamus, oxygen homeostasis, inflammation

## Abstract

Heat stress (HS) is devastating to poultry production sustainability due its detrimental effects on performance, welfare, meat quality, and profitability. One of the most known negative effects of HS is feed intake depression, which is more pronounced in modern high-performing broilers compared to their ancestor unselected birds, yet the underlying molecular mechanisms are not fully defined. The present study aimed, therefore, to determine the hypothalamic expression of a newly involved pathway, hypoxia/oxygen homeostasis, in heat-stressed broiler-based research lines and jungle fowl. Three populations of broilers (slow growing ACRB developed in 1956, moderate growing 95RB from broilers available in 1995, and modern fast growing MRB from 2015) and unselected Jungle fowl birds were exposed to cyclic heat stress (36°C, 9 h/day for 4 weeks) in a 2 × 4 factorial experimental design. Total RNAs and proteins were extracted from the hypothalamic tissues and the expression of target genes and proteins was determined by real-time quantitative PCR and Western blot, respectively. It has been previously shown that HS increased core body temperature and decreased feed intake in 95RB and MRB, but not in ACRB or JF. HS exposure did not affect the hypothalamic expression of HIF complex, however there was a line effect for HIF-1α (*P* = 0.02) with higher expression in JF under heat stress. HS significantly up regulated the hypothalamic expression of hemoglobin subunits (HBA1, HBBR, HBE, HBZ), and HJV in ACRB, HBA1 and HJV in 95RB and MRB, and HJV in JF, but it down regulated FPN1 in JF. Additionally, HS altered the hypothalamic expression of oxygen homeostasis- up and down-stream signaling cascades. Phospho-AMPK^Thr172^ was activated by HS in JF hypothalamus, but it decreased in that of the broiler-based research lines. Under thermoneutral conditions, p-AMPK^Thr172^ was higher in broiler-based research lines compared to JF. Ribosomal protein S6K1, however, was significantly upregulated in 95RB and MRB under both environmental conditions. HS significantly upregulated the hypothalamic expression of NF-κB2 in MRB, RelB, and TNFα in ACRB, abut it down regulated RelA in 95RB. The regulation of HSPs by HS seems to be family- and line-dependent. HS upregulated the hypothalamic expression of HSP60 in ACRB and 95RB, down regulated HSP90 in JF only, and decreased HSP70 in all studied lines. Taken together, this is the first report showing that HS modulated the hypothalamic expression of hypoxia- and oxygen homeostasis-associated genes as well as their up- and down-stream mediators in chickens, and suggests that hypoxia, thermotolerance, and feed intake are interconnected, which merit further in-depth investigations.

## Introduction

Heat stress (HS) negatively affects performance, health, and welfare of birds, thereby imperiling the poultry production sustainability (1). From a thermophysiological point of view, HS is a thermoregulatory imbalance that occurs when the animals metabolic heat production exceeds their own capacity to dissipate body heat to the surrounding environment (2). Chickens are homeothermic endotherms that have the ability to generate deep body heat, and adopt evolutionary conserved strategies to maintain a relatively high and constant body temperature (3). The most known and prominent response to HS is feed intake depression (4–7), however the underlying molecular mechanisms are still not fully defined.

Feed intake regulation is highly conserved across animals and a series of tightly integrated and interconnected neural mechanisms are involved (8, 9). First brain lesioning, stimulation, and electrophysiological studies evolved the dual center model and identified the hypothalamus, with the satiety and hunger centers, as a major site controlling appetite, feed intake, and body weight (10–12). In search for this central model's components and with the advances and revolutions in molecular techniques, subsequent studies over ensuing years have identified numerous (an)orexigenic neuropeptides (13–16). In a recent study, it was reported that chronic cyclic heat stress in broilers did not alter the hypothalamic expression of the classical neuropeptides, namely neuropeptide Y (NPY), agouti-related peptide (AgRP), proopiomelanocortin (POMC), and cocaine- and amphetamine-regulated transcript (CART) (17), suggesting potential involvement of other central pathways.

In their study, Zhang et al. (18) made a breakthrough by demonstrating a new function for hypoxia-inducible factor (HIF) signaling in the hypothalamus and its ability to regulate feeding behavior, body weight, and metabolic homeostasis. In fact, ablation of HIF in the arcuate nucleus POMC neurons resulted in hyperphagia and increased fat deposition (18). Furthermore, factor inhibiting HIF (FIH)-knock out mouse exhibited hypermetabolic phenotype, with greatly increased food intake, energy expenditure, and ventilation rate (19). Interestingly, the energy sensor AMP-activated kinase (AMPK) and the nutrient sensor mechanistic target of rapamycin (mTOR) signaling pathways were both found to converge on HIF (18). Heat shock proteins (HSPs), nuclear factor κB (NF-κB), and IL-6, on the other hand, were found to be downstream mediators of HIF (20–22). AMPK and mTOR are well-known regulators of feed intake (23, 24). Similarly, NF-κB, HSPs, and IL-6 have been shown to regulate feed intake and energy homeostasis in mammals (25–27). Together, these data indicate that the two apparently disparate processes controlled by HIF, i.e., sensing/coordination of oxygen homeostasis and food intake regulation may be linked.

Heat-stressed birds divert blood to the periphery (skin) to maximize sensible heat loss. These results in reduced blood flow to internal organs such as the brain (28, 29) and leads to hypoxia-like state. It is our hypothesis that the central HIF signaling pathway is modulating feed intake regulation. The current study sets to determine the expression profile of hypothalamic HIF complex as well as its upstream regulators (PI3K, AMPK, mTOR) and downstream mediators (NF-κB, IL6, HSPs) in heat-stressed jungle fowl and three experimental broiler chicken lines established at 20-year intervals.

## Materials and Methods

### Chicken Populations

The chicken populations used in the present study are maintained and hatched at the poultry facilities of the University of Arkansas. Each generation was randomly mated with the exception of full and half siblings to limit inbreeding. The Athens Canadian Random Bred (ACRB), a slow-growing line, was established from commercially available broiler populations in 1956 (30). The moderate-growing 1995 random bred (95RB) represented the composite of broiler populations available in 1995 (31). The fast-growing modern random bred (MRB) was developed from the composite of high-yielding commercial broilers available in 2015 (31). Lastly, jungle fowl (JF), namely the wild forebear of domestic chicken (32), was the fourth population. This study was approved by the Animal Care and Use Committee of the University of Arkansas with protocol numbers 18083 and 16084, while its procedures conformed to the guide for the care and use of laboratory animals of the National Institutes of Health.

### Animal Husbandry and Experimental Design

A total of 600 newly hatched male chicks (*n* = 150/population) were wing-tagged with a number code and randomly allocated to 12 environmentally controlled chambers divided into two pens of the same size (25 birds/pen). A factorial experimental design (2 × 4), having environmental temperature and line (population) as the main factors and pens as replicates, was used. All pens were matched with regard to feeders, drinkers, and wood shavings as bedding material. Daily, birds were manually fed and watered *ad libitum*. Commercially available starter (0–28 days) and finisher diets (29–56 days) were provided. The artificial photoperiod was 23L:1D during the first 7 days, while 20L:4D for the remainder of the trial. The environmental temperature was gradually decreased in all chambers as follows: 32°C (0–3 days), 31°C (4–6 days), 29°C (7–10 days), 27°C (11–14 days), and 25°C (15–28 days). From 29 to 56 days, half of the chambers were constantly kept at thermoneutral conditions (TN, 25°C), while the other half subjected to cyclic heat stress (HS, 36°C, 0900–1800 h) as detailed in our recent study (17). The relative humidity was between 40 and 60% (31).

### Hypothalamic Samples Collection

As previously described (17), two birds per pen (i.e., six birds/combination of environmental temperature and line) were randomly chosen and euthanized *via* cervical dislocation. Hypothalamic samples were collected and treated according to the procedures reported by Piekarski and co-workers (33). Briefly, the entire brain was extracted from the skull and immersed in 2-methylbutane (Sigma, St, Louis, MO) in dry ice for 60 s to provide firmness needed for the hypothalamic dissection. This was done in accordance with the chick brain's stereotaxic atlas published by Kuenzel and Masson (34). Brains were placed on a cold metal plate with the ventral side visible. A forward cut was performed at the corticoseptomesencephalic tract (otherwise known as septopalliomesencephalic tract), while a posterior cut was also performed at the third oculomotor nerve. Two cuts were done bilaterally 2 mm from the brain midline. Lastly, a 5 mm cut from the brain base was performed dorsally to obtain the whole hypothalamus.

### RNA Isolation, Reverse Transcription, and Real-Time Quantitative PCR

RNA isolation, reverse transcription, and real-time quantitative PCR were carried out as previously reported by our group (35–37). Briefly, total RNA was isolated from hypothalamic samples (*n* = 48) with Trizol reagent (Life Technologies, Carlsbad, CA) according to the manufacturer's instructions. Purity and concentrations of RNA were evaluated through Take3 micro-volume plate and Synergy HT multimode microplate reader (BioTek, Winooski, VT). One sample belonging to JF-HS group was omitted due to poor RNA quality. RNA samples were RQ1 RNase-free DNase treated (Promega, Madison, WI), and 1 μg RNA was reverse transcribed *via* qScript cDNA Synthesis Kit (#95048-100, Quanta Biosciences, Gaithersburg, MD). The reverse transcription reaction was done at 42°C for 30 min, followed by an incubation of 5 min at 85°C. Real-time quantitative PCR (Applied Biosystems 7500 Real-Time PCR System) was performed in a total reaction of 12.5 μl with 2.5 μl of cDNA, 0.5 μl of forward and reverse primers, and SYBR Green Master Mix (ThermoFisher Scientific, Rockford, IL). The chicken-specific oligonucleotide primers used for hemoglobin subunits (HBA1, HBBR, HBM, HBZ, and HBE), Ferritin Heavy Chain 1 (FTH1), Ferroportin-1(FPN1), hephaestin (HEPH), hemojuvelin (HJV), Hypoxia-inducible factor-1α (HIF-1α), Egl-9 Family Hypoxia Inducible Factor 1(EGNL1), protein kinase B (AKT), phosphatidylinositol 3-kinase alpha (PI3Kα), AMP-activated protein kinase (AMPK) subunits (α1/2, β1/2, and γ1–3), interleukin 6 (IL-6), tumor necrosis factor alpha (TNFα), heat-shock proteins (HSP27, HSP60, HSP70, and HSP90), and ribosomal 18S as a housekeeping gene were previously published (6, 38–40). The sequences of the other oligonucleotide primers used are summarized in Table 1. The qPCR cycling conditions were 50°C for 2 min and 95°C for 10 min, followed by 40 cycles of a two-step amplification program (95°C for 15 s and 58°C for 60 s). According to Schmittgen and Livak (41), relative expression of target genes was computed through the 2^−ΔΔCT^ method with 18S rRNA as the housekeeping gene and JF-TN group as calibrator.

**Table 1:** List of qPCR chicken-specific oligonucleotide primers.

**Gene**	**Accession number^**†**^**	**Primer sequence (5^′^ → 3^′^)**	**Orientation^§^**	**Product size (bp)**
HIF-2α	NM_204807.3	CCAGTGCGTTCTCCCAACAT	F	66
		GCCTCGTTGCCCCAAAC	R	
PI3Kβ	NM_001031311.1	TGCCTCCTGCCGTGACA	F	61
		TCAGCACCGATCTGTGAATCC	R	
PI3Kδ	NM_001012696.2	TGACCAATATCCACAAGCTTTGG	F	69
		GCCACGTCTTCATGCTTGTTC	R	
NF-κB1	NM_205134.2	CAGTCAACGCAGGACCTAAAGA	F	65
		TGTGACGTGAAGTATTCCAAGGTT	R	
NF-κB2	NM_204413.2	AGATCTCGCGGATGGACAAG	F	92
		CTCAATGTCATCTTTCTGCACCTT	R	
RelA	NM_001396038.1	CGCTGCGTGCACAGTTTC	F	61
		CTTCCAGTTCCCGTTTCTTCAC	R	
RelB	XM_003643092.5	CCACGGCGCTAATAATTTGC	F	60
		GAAGGGCATTGCATGCATT	R	

*Accession number refers to GenBank (National Center for Biotechnology Information – NCBI)*.

§ *F, forward; R, reverse*.

*HIF, hypoxia-inducible factor; PI3K, phosphatidylinositol 3-kinase; NF-κB, nuclear factor kappa-light-chain-enhancer of activated B cells*.

### Protein Isolation and Western Blot Analysis

The analytical procedures illustrated here were performed along the lines of our previously published article (42). Hypothalamic samples were homogenized in lysis buffer (10 mmol/L Tris base, pH 7.4; 150 mmol/L NaCl; 1 mmol/L EDTA;0.1% Triton X-100;0.5% Nonidet P-40; protease and phosphatase inhibitors) using glass beads and Bullet Blender Storm (NextAdvance, Averill Park, NY). Total protein concentration was assessed with Synergy HTX (BioTek, Winooski, VT) and Bradford assay kit (#5000006, Bio-Rad Laboratories, Inc., Hercules, CA). Proteins (80 μg) were run on 4%−12% Novex Bis–Tris gels (Life Technologies, Grand Island, NY) and then transferred to polyvinylidene difluoride membranes. Membranes were blocked using TBS with 5% non-fat milk and Tween 20 at RT for 1 h. Later, membranes were washed with TBS and incubated overnight with primary antibodies (1:500–1:1,000) at 4°C. The primary antibodies used were rabbit polyclonal anti-phospho AMPKα1/2^Thr172^ (#2531, Cell Signaling Technology, Danvers, MA), anti-AMPKα1/2 (#2795, Cell Signaling Technology, Danvers, MA), anti-HSP90 (#PA5-17610, Pierce Thermo Scientific, Rockford, IL), goat polyclonal anti-HSP60 (#sc-1052, Santa Cruz Biotechnology, Dallas, TX), mouse monoclonal anti-HSP70 (#MAI-91159, Pierce Thermo Scientific, Rockford, IL) and rabbit anti-GAPDH (Santa Cruz Biotechnology, Dallas, TX). After a wash with TBS, HRP-conjugated secondary antibodies (1:5,000) were added to 5% non-fat milk in TBS and Tween 20 and incubated with the membranes at RT for 1 h. Protein signals were visualized through chemiluminescence with Super ECL (ABP BioSciences, Beltsville, MD), while images captured using FluorChem M MultiFluor System (ProteinSimple, Santa Clara, CA). Image acquisition and analysis were performed with AlphaView software ver. 3.4.0 (ProteinSimple, Santa Clara, CA). Relative protein levels were presented as a ratio of phospho protein/Pan protein or total protein/GAPDH.

### Statistics

Data were analyzed by two-way ANOVA with environmental temperature and line as fixed factors, while the sampled animal as experimental unit. If ANOVA revealed significant effects, the means were compared by Tukey multiple range test using the Graph Pad Prism version 6.00 for Windows (Graph Pad Software, La Jolla California, USA). Differences were considered significant at *P* < 0.05.

## Results

### Expression Profile of Hypothalamic Hypoxia- and Oxygen Homeostasis-Related Genes in Heat-Stressed Broilers and Their Ancestor JF

**Figure 1:**
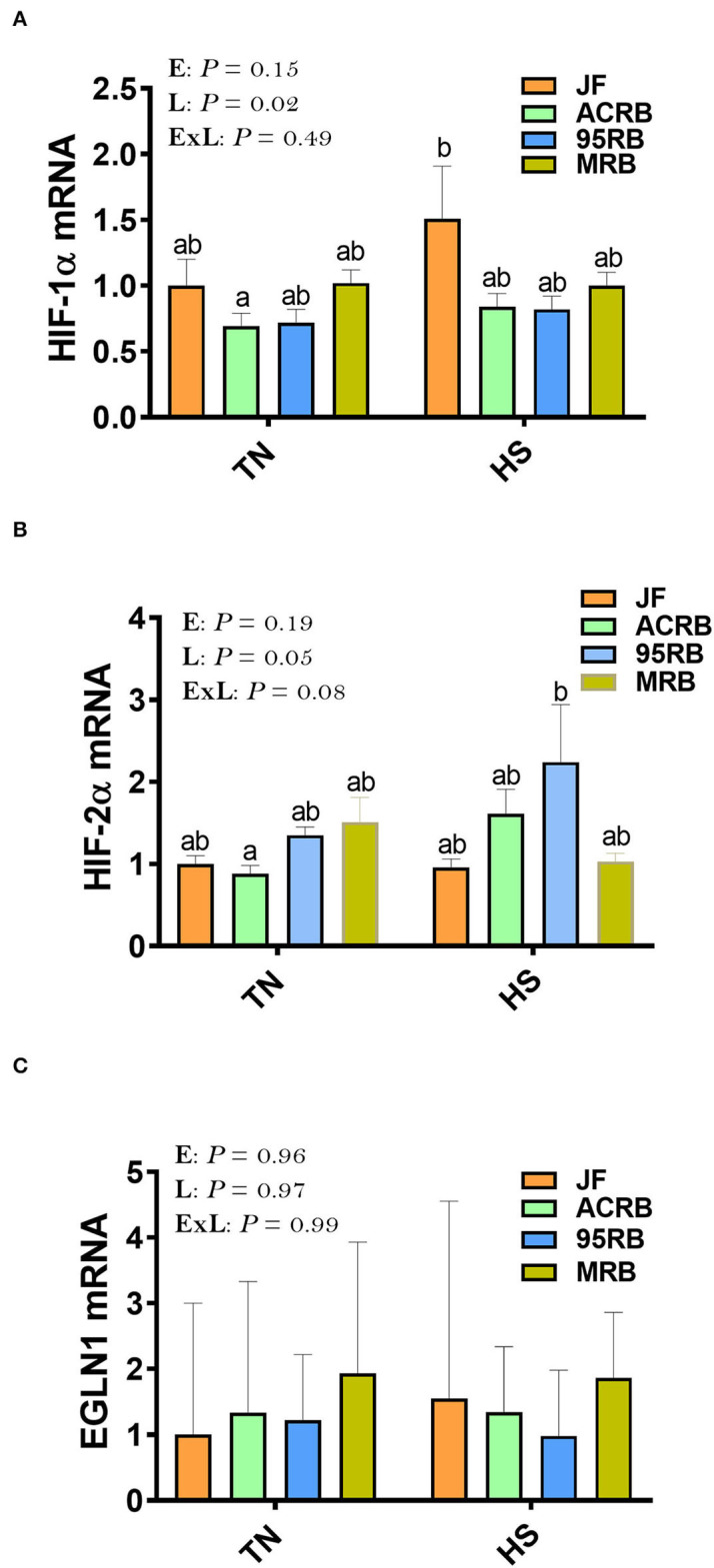
Effect of HS on hypothalamic expression of HIF complex in three modern broilers and their ancestor JF. **(A)** HIF-1α, **(B)** HIF-2α, and **(C)** EGLN1. RNA was extracted from hypothalamic tissues and analyzed by qPCR using 2^−ΔΔCT^ method (41). Gene expression data are mean ± SEM (*n* = 6/group). Different letters indicate significant difference at *P* < 0.05. 95RAN, 1995 random bred; ACRB, Athens Canadian Random Bred; E, environment; EGNL1, Egl-9 Family Hypoxia Inducible Factor 1; ExL, interaction between E and L; HIF, hypoxia-inducible factor; HS, heat stress (36°C); JF, jungle fowl; L, line; MRB, modern random bred; TN, thermoneutral (25°C).

**Figure 2:**
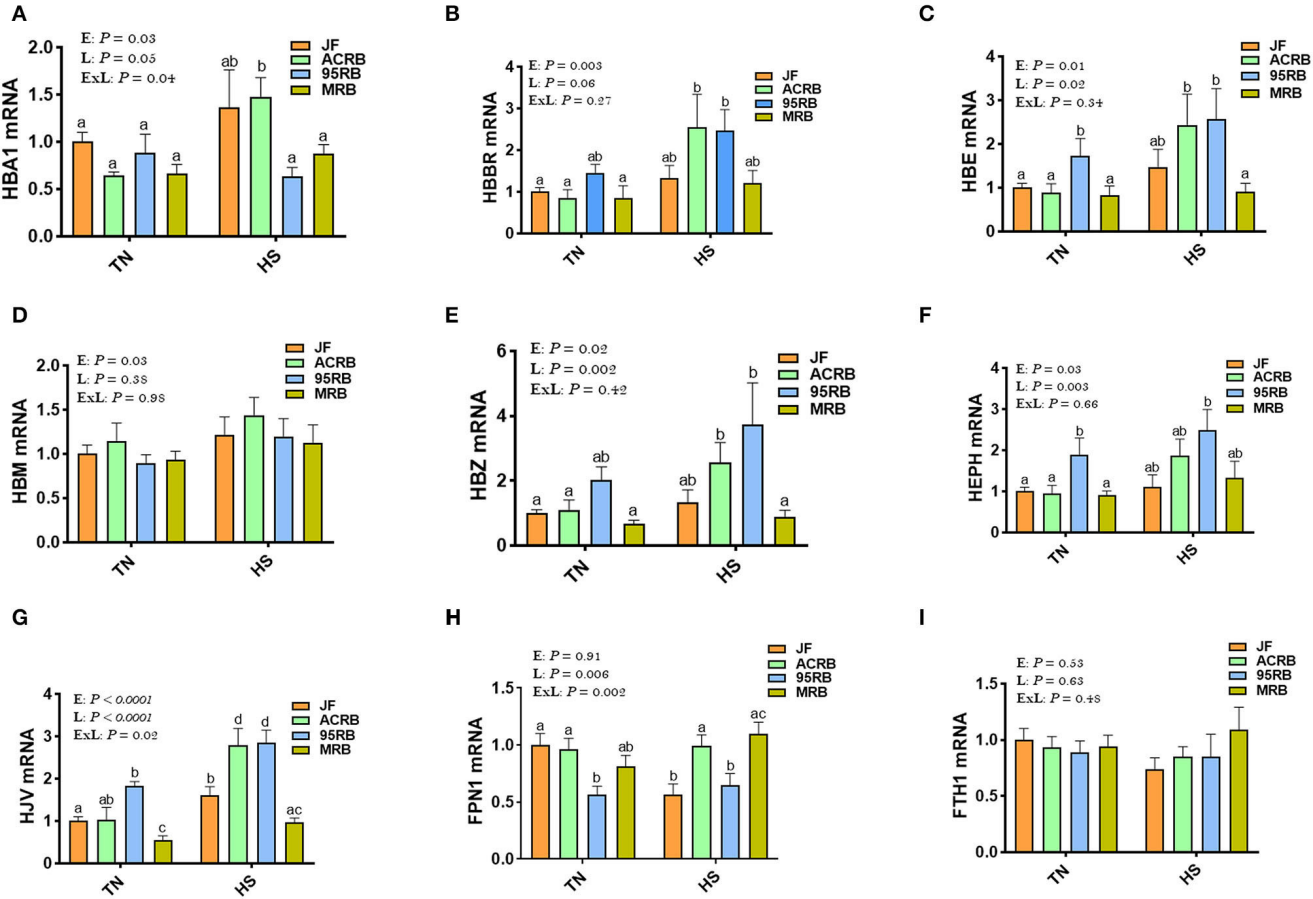
Effect of HS on hypothalamic expression of oxygen homeostasis-associated genes in three modern broilers and their ancestor JF. RNA was extracted from hypothalamic tissues and analyzed by qPCR using 2^−ΔΔCT^ method. Data for gene expression of HBA1 **(A)**, HBBR **(B)**, HBE **(C)**, HBM **(D)**, HBZ **(E)**, HEPH **(F)**, HJV **(G)**, FPN1 **(H)**, and FTH1 **(I)** are mean ± SEM (*n* = 6/group). Different letters indicate significant difference at *P* < 0.05. 95RAN, 1995 random bred; ACRB, Athens Canadian Random Bred; E, environment; ExL, interaction between E and L; FTH1, Ferritin Heavy Chain 1; FPN1, Ferroportin-1; HBA1, hemoglobin Subunit Alpha; HBBR, hemoglobin beta, subunit rho; HBE, hemoglobin E; HBM, hemoglobin Subunit Mu; HBZ, hemoglobin Subunit Zeta; HEPH, hephaestin; HJV, hemojuvelin; HS, heat stress (36°C); JF, jungle fowl; L, line; MRB, modern random bred; TN, thermoneutral (25°C).

Exposure to chronic cyclic HS did not affect the hypothalamic expression of HIF-1α, HIF-2α, or EGLN1 (Figures 1A–C). However, there was a significant line effect (*P* = 0.02) for hypothalamic HIF-1α with higher levels in JF compared to the three other broiler lines under HS conditions (Figure 1A). The hypothalamic expression of HIF-2α and EGLN1 did not differ between the studied lines under thermoneutral or HS conditions (Figures 1B,C). For the oxygen homeostasis-associated genes, there was a significant line effect for HBA1, HBBR, HBE, HBM, HBZ, HEPH, HJV, and FPN1 with a higher expression found in ACRB-hypothalamus for HBA1, HBRR, HBE, HBZ, HJV, and FPN1 under HS conditions (Figures 2A–E,G,H). The 95RB birds exhibited elevated abundance of hypothalamic HBE, HEPH, and HJV mRNA under both environmental conditions (Figures 2C,F,G) and HBZ under HS conditions (Figure 2E). Heat stress up regulated the hypothalamic expression of HBA1, HBBR, HBE, HBZ, and HJV in ACRB (Figures 2A–C,G), HBZ and HJV in 95RB (Figures 2E,G), and HJV in JF (Figure 2G), but it down regulated that of FPN1 in JF (Figure 2H).

### Effect of HS on Hypothalamic AMPK-mTOR Pathway in Broiler-Based Research Lines and Their Ancestor JF

**Figure 3:**
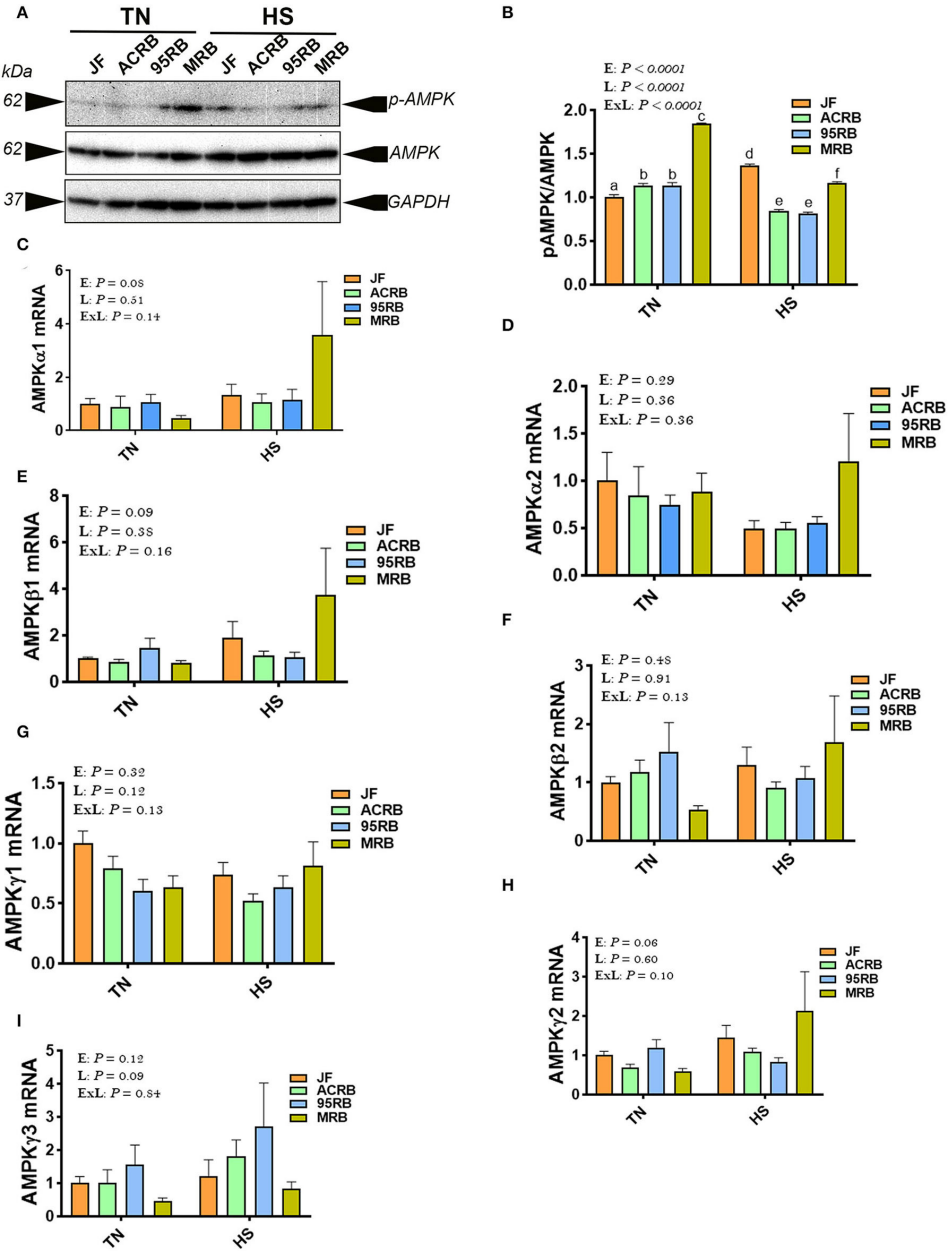
Effect of HS on hypothalamic expression of AMPK pathway in three modern broilers and their ancestor JF. Protein and RNA were extracted from hypothalamic tissues and analyzed by western blot **(A,B)** and qPCR **(C–I)**, respectively. Gene expression was determined by qPCR using 2^−ΔΔCT^ method and data are mean ± SEM (*n* = 6/group). Protein expression was presented as ratio of p-AMPK^Thr172^/pan AMPK **(A,B)**. Protein was analyzed *via* AlphaVIew software and is expressed as mean ± SEM (*n* = 3/group) with one representative blot shown **(A)**. Different letters indicate significant difference at *P* < 0.05. 95RAN, 1995 random bred; ACRB, Athens Canadian Random Bred; AMPK, AMP-activated protein kinase; E, environment; ExL, interaction between E and L; HS, heat stress (36°C); JF, jungle fowl; L, line; MRB, modern random bred; TN, thermoneutral (25°C).

**Figure 4:**
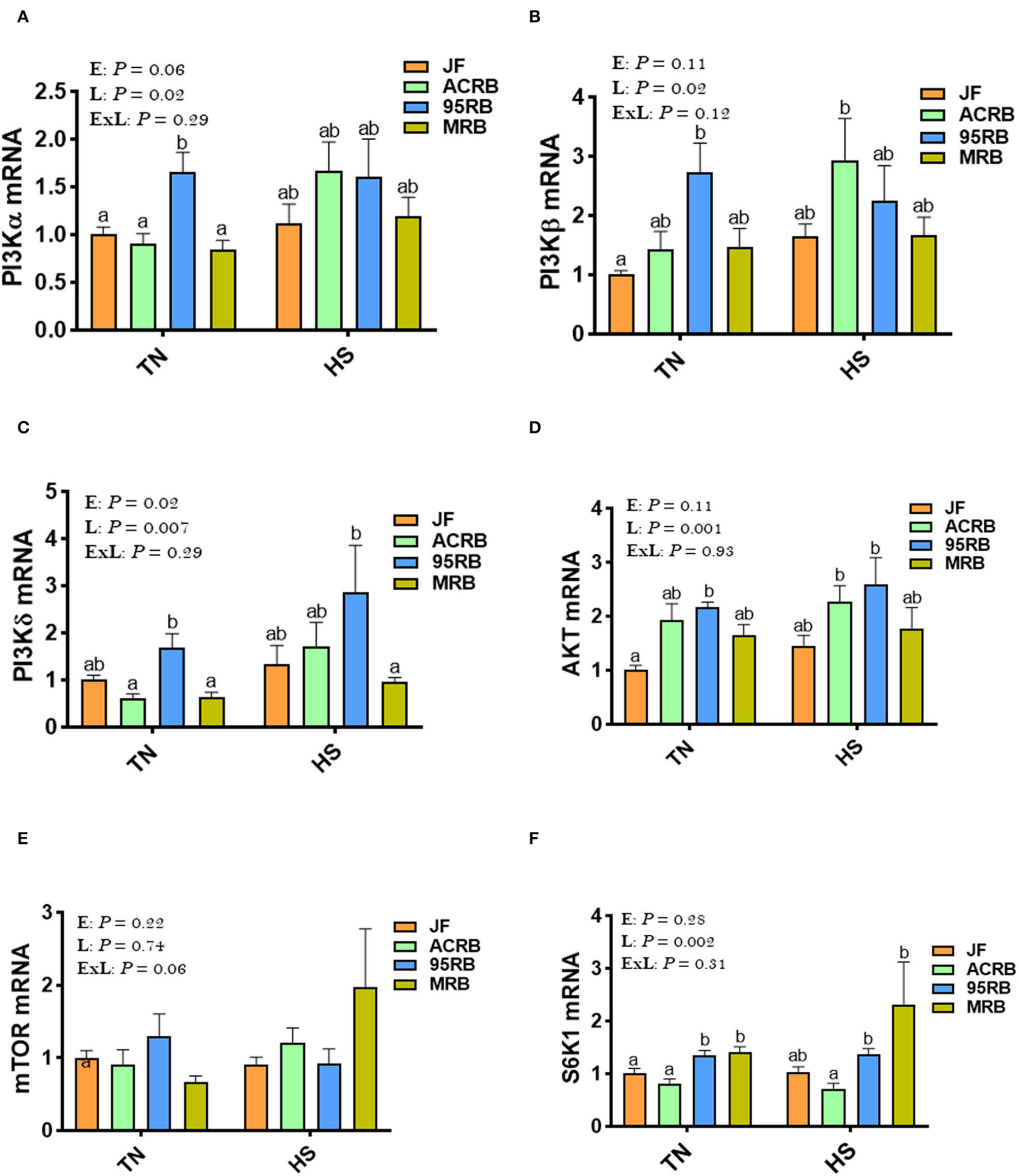
Effect of HS on hypothalamic expression of PI_3_K-mTOR-S6K1 pathway in three modern broilers and their ancestor JF. RNA was extracted from hypothalamic tissues and analyzed by qPCR using 2^−ΔΔCT^ method. Data for gene expression of PI3Kα **(A)**, PI3Kβ **(B)**, PI3Kγ **(C)**, AKT **(D)**, mTOR **(E)**, and S6K1 **(F)** are mean ± SEM (*n* = 6/group). Different letters indicate significant difference at *P* < 0.05. 95RAN, 1995 random bred; ACRB, Athens Canadian Random Bred; AKT, protein kinase B; E, environment; ExL, interaction between E and L; HS, heat stress (36°C); JF, jungle fowl; L, line; MRB, modern random bred; mTOR, mechanistic target of rapamycin; PI3K, phosphatidylinositol 3-kinase; S6K1, Ribosomal protein S6 kinase beta-1; TN, thermoneutral (25°C).

Heat stress increased the phosphorylated levels of AMPKα1/2 at Thr^172^ site in JF hypothalamus, but it reduced it in broiler-based research lines compared to TN conditions (Figures 3A,B). Under TN conditions, MRB exhibited higher levels of hypothalamic p-AMPKα1/2^Thr172^, followed by 95RB, ACRB, and JF (Figures 3A,B). Under heat stress conditions, however, JF hypothalamus contained elevated levels of p-AMPKα1/2^Thr172^, followed by MRB, ACRB, and 95RB, which resulted in a significant environment x line interaction (*P* < 0.0001; Figures 3A,B). The gene expression of hypothalamic AMPKα1, AMPKα2, AMPKβ1, AMPKβ2, AMPKγ1, AMPKγ2, and AMPKγ3 did not differ between the studied lines under all environmental conditions (Figures 3C–I). Analyzing the upstream regulators of HIF, the qPCR data showed that heat stress did not affect the hypothalamic expression of PI3K, AKT, mTOR, and S6K1 (Figures 4A–F). Under TN conditions, 95RB exhibited higher levels of hypothalamic PI3Kα and PI3Kγ compared to other lines (Figures 4A,C). The hypothalamic expression of S6K1 gene was found to be upregulated in modern 95RB and MRB broilers compared to JF and ACRB under both environmental conditions (Figure 4F).

### Effect of HS on the Expression of Hypothalamic NF-κB Complex, TNFα, and IL-6 in Broiler-Based Research Lines and Their Ancestor JF

**Figure 5:**
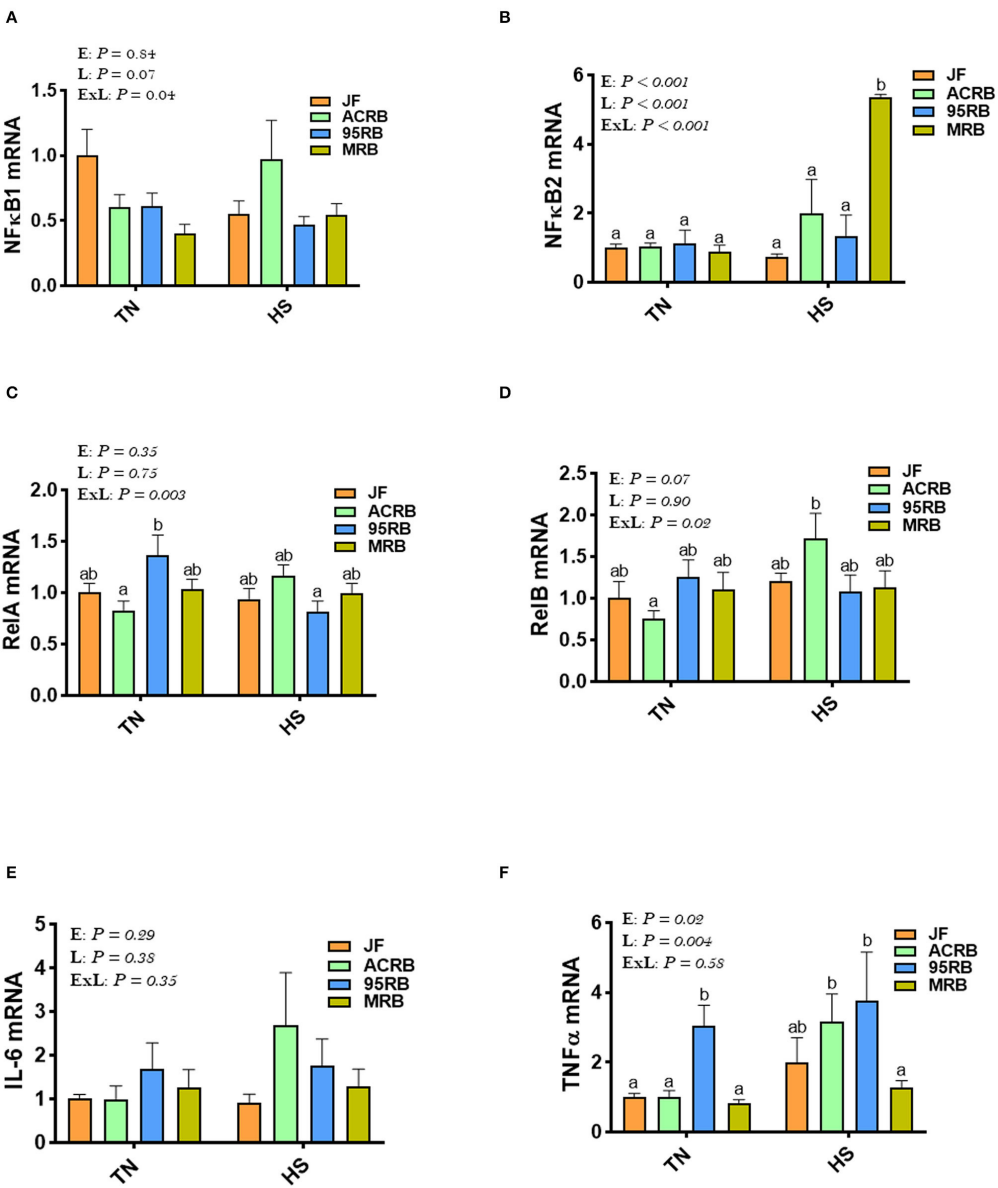
Effect of HS on hypothalamic expression of NF-κB pathway, TNFα, and IL-6 in three modern broilers and their ancestor JF. RNA was extracted from hypothalamic tissues and analyzed by qPCR using 2^−ΔΔCT^ method. Data for gene expression of NF-κB1 **(A)**, NF-κB2 **(B)**, RelA **(C)**, RelB **(D)**, IL-6 **(E)**, and TNFα **(F)** are mean ± SEM (*n* = 6/group). Different letters indicate significant difference at *P* < 0.05. 95RAN, 1995 random bred; ACRB, Athens Canadian Random Bred; E, environment; ExL, interaction between E and L; HS, heat stress (36°C); IL-6, interleukin 6; JF, jungle fowl; L, line; MRB, modern random bred; NF-κB, Nuclear Factor Kappa B; RelA/B, v-rel avian reticuloendotheliosis viral oncogene homolog A/B; TN, thermoneutral (25°C); TNFα, tumor necrosis factor.

Heat stress up regulated the hypothalamic expression of NF-κB2 gene in MRB, and RelB and TNFα in ACRB, but it down regulated that of relA in 95RB compared to TN conditions (Figures 5B–D,F). Under TN conditions, the 95RB exhibited higher levels of RelA and TNFα compared to the other lines (Figures 5C,D). Under HS conditions, MRB manifested elevated mRNA abundances of NF-κB2 (Figure 5B), and ACRB and 95RB exhibited higher levels of TNFα (Figure 5F). The hypothalamic expression of NF-κB1 and IL-6 did not differ between all studied lines under both environmental conditions (Figures 5A,E).

### Effect of HS on the Expression of Hypothalamic HSPs in Broiler-Based Research Lines and Their Ancestor JF

**Figure 6:**
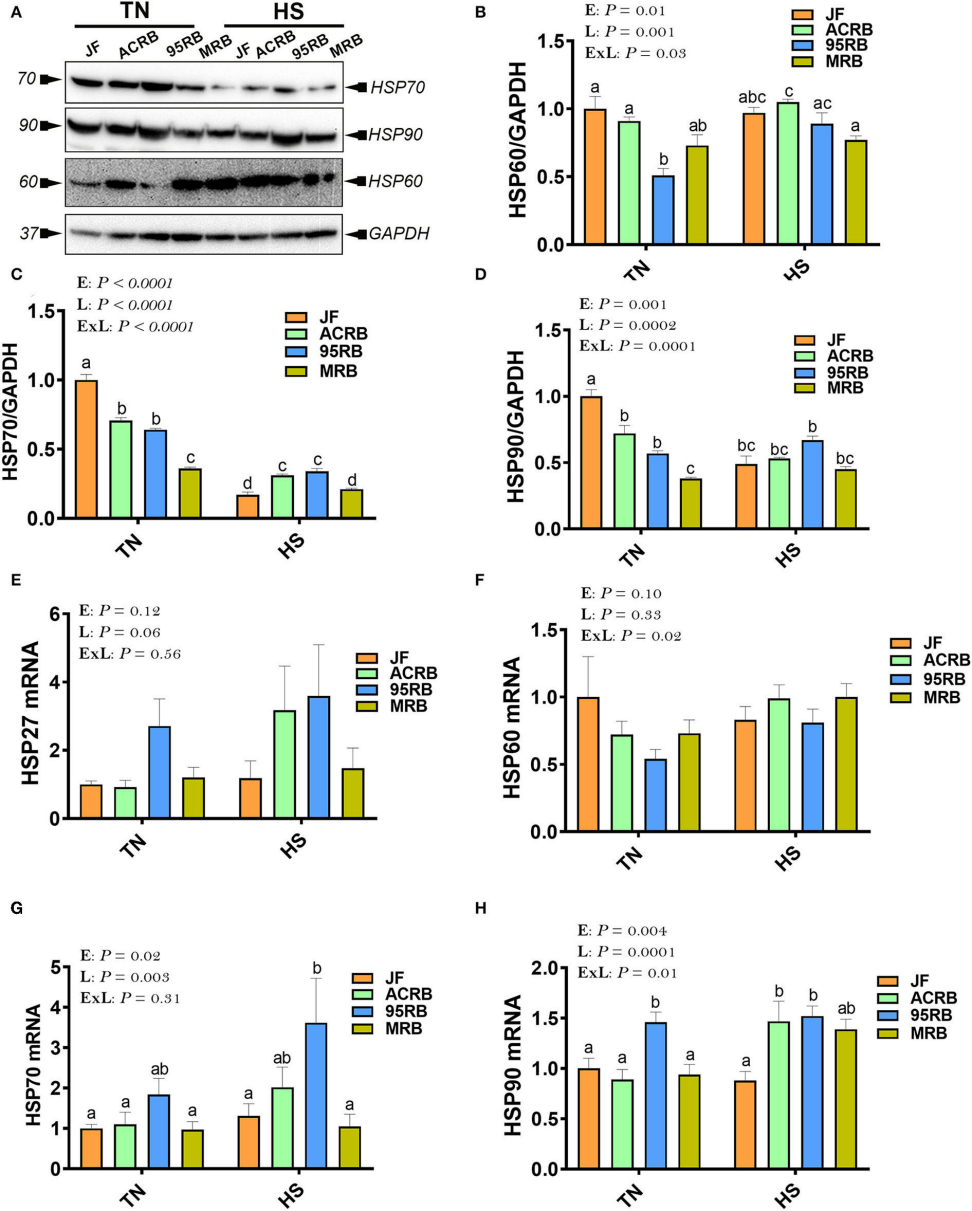
Effect of HS on hypothalamic expression of HSPs in three modern broilers and their ancestor JF. Protein and RNA were extracted from hypothalamic tissues and analyzed by western blot **(A–D)** and qPCR **(E–H)**, respectively. Gene expression was determined by qPCR using 2^−ΔΔCT^ method and data are mean ± SEM (*n* = 6/group). Protein expression was presented as target protein/GAPDH **(A–D)**. Protein was analyzed *via* AlphaVIew software and is expressed as mean ± SEM (*n* = 3/group) with one representative blot shown **(A)**. Different letters indicate significant difference at *P* < 0.05. 95RAN, 1995 random bred; ACRB, Athens Canadian Random Bred; E, environment; ExL, interaction between E and L; HS, heat stress (36°C); HSP, heat shock protein; JF, jungle fowl; L, line; MRB, modern random bred; TN, thermoneutral (25°C).

Immunoblot analyses showed that HS exposure increased the protein levels of HSP60 in the hypothalamus of ACRB and 95RB (Figures 6A,B), and reduced that of HSP70 in all studied lines (Figures 6A,C) compared to their TN-counterparts. The hypothalamic protein levels of HSP90 were reduced by HS only in JF (Figure 6D). At the mRNA levels, HS up regulated the hypothalamic expression of HSP90 in both ACRB and 95RB birds (Figure 6H), however it did not elicit any change to HSP27, HSP60, or HSP70 mRNA abundances (Figures 6E–G). Under TN conditions, JF exhibited higher levels of HSP protein levels followed by ACRB, 95RB, and MRB (Figures 6A–D). Under HS conditions, the protein levels of HSP60 did not change much between the lines, however HSP90 mRNA was higher in the modern lines, ACRB, 95RB, MRB, compared to JF (Figure 6H).

## Discussion

Heat stress is one of the most challenging stressors to poultry production sustainability due to its adverse effects on growth performances, welfare, health, meat quality, and consequently profitability (1, 43–48). The above-mentioned negative effects were found to be more pronounced in high-performing broiler-based research lines when compared to their ancestor JF (31), because of their high metabolic rate and elevated circulatory and respiratory demands (49, 50). As extensively reported, one of the prominent effects of heat stress is reduced feed intake to decrease heat gain from digestion and metabolism (6, 51, 52), however the underlying molecular mechanisms for these heat stress responses are not fully understood.

As the brain is the main site controlling energy intake and expenditure (53–56), and as feed intake and oxygen/hypoxia-sensing are interconnected, we sought to determine here the effect of cyclic heat stress on hypothalamic hypoxia- and oxygen-associated pathways in three broiler-based research lines and their ancestor JF. Although HS did not affect the hypothalamic expression of HIF complex (HIF-1α, HIF-2α, EGNL1), HIF-1α mRNA abundances were higher in JF hypothalami compared to the broiler-based research lines, particularly under heat stress conditions. HIF-1 is a dimeric transcription factor complex (57) that increases vascularization in hypoxic area and thereby plays an integral role in the body's homeostasis and response to low oxygen (58, 59). As heat stress has been shown to induce hypoxia in internal organs, including brain (60) and as JF birds were found to be more thermotolerant compared to the broiler-based research lines (61), our data suggest that the increased expression of hypothalamic HIF-1α might improve the overall capacity of central O_2_ transport and in turn enhance hypoxia tolerance in JF chickens. In support of this hypothesis, we have previously shown that heat stress increased core body temperature of modern broilers but not that of JF (31), indicating that JF have better ability to withstand high ambient temperature than modern broilers. Furthermore, modern broilers are heavier than JF and a negative association between body weight/size and heat tolerance has been well documented (62, 63). Although it is generally admitted that the O_2_ transport pathway of birds has several unique characteristics that help support energetic activity and aerobic metabolism during hypoxia (64), modern broilers exhibit high O_2_ requirements because of their strenuous digestion, high metabolism, and fast growth, without concomitant development of cardiovascular/respiratory system efficiency (65, 66). In fact, several elegant studies have shown that fast-growing broilers have thicker respiratory membrane, lower O_2_ transfer efficiency, and lower hemoglobin oxygenation capability (50, 65). Moreover, a relationship between heat tolerance and oxygen homeostasis in chicken has been reported (67). Whilst further mechanistic studies are warranted, our data suggest that hypothalamic HIF-1α might be involved in the regulation of feed intake, hypoxia- and thermo-tolerance in chickens.

The acclimatization and adaptive erythropoietic response to hypoxia generally involves increases in hematocrit and hemoglobin concentrations (64, 68, 69); however, our qPCR analyses did not show a significant alteration in the hypothalamic expression of oxygen homeostasis-associated genes, except a significant down regulation of FPN1 in heat-stressed JF, upregulation of HJV in JF, ACRB, and 95RB under HS conditions, and a significant increase of HBA1, HBBR, HBE, and HBZ in heat-stressed ACRB. This apparent inconsistency is unexpected and does not support the abovementioned adaptive response to hypoxia, at least for the JF birds. However, although the aforesaid conclusion is valid, it must be considered cautiously and supported by further evidence for several reasons. First, we measured here only gene expression, and hemoglobin saturation and its O_2_-binding properties were not measured, which is a limitation in this study. Second, the roles of the hypothalamic oxygen genes in feed intake regulation are still unknown in chickens. As calorie restriction has been shown to regulate FPN1 expression (70, 71), and as iron has been reported to control feed intake in mammals (72), it is therefore reasonable to hypothesize that dysregulation of FPN1 in heat-stressed JF might play a role in heat stress adaptation and feed intake control, however further in-depth investigations are required.

Aside its role in oxygen sensing, HIF regulates many biological processes, including inflammation (73, 74) and glucose metabolism (75, 76), both of which have been reported to be affected by heat stress (77, 78) and regulate feed intake (79, 80). In our experimental conditions, there was an upregulation of hypothalamic NF-κB2 by heat stress only in MRB, RelB, and TNFα in ACRB, and a down regulation of RelA in 95RB. The activation of NF-κB in the hypothalamus has been shown to recruit pro-inflammatory microglia and promote appetite and overeating in mammals (81, 82), suggesting that NF-κB2 might play a role in feed intake regulation in MRB. However, this effect seems to be only during heat stress as the hypothalamic expression of NK-κB2 was not affected in TN conditions. NF-κB has been shown to be regulated by heat stress (83) and induces the expression of various pro-inflammatory genes, such as the cytokines TNFα and IL-6 (84), which have been also reported to regulate feeding behavior (79, 85, 86). The lack of hypothalamic TNFα- and IL6-modulation in the present study proposes that this cytokine pathway was not involved. It is worth noting however that we, again, measured only gene expression and not proteins (the motor elements and workhorse of the cell) because of lack of cross reactive antibodies.

To gain further insights, we next assessed the hypothalamic expression of AMPK [the energy sensor (87)] and mTOR [the nutrient sensor (88)] pathway, because both have been shown to regulate HIF expression and feeding behavior (18, 23, 24, 89). AMPK is a phylogenetically conserved serine/threonine heterotrimeric kinase consisting of catalytic alpha subunit (two isoforms α1 and α2), and regulatory beta (two isoforms β1 and β2), and gamma subunits (three isoforms γ1, γ2, and γ3), that sensing energy levels by detecting elevation in the AMP/ATP ratio (90, 91). Under energy stress (depletion), AMPK is fully activated through binding of AMP to the γ-subunit and induction of Thr172 phosphorylation in the α-subunit, and thereby promotes catabolic pathways to restore energy balance (92). The increased levels of hypothalamic p-AMPK^Thr172^ in broiler-based research lines compared to JF in our experimental conditions is not surprising and indicate that these lines had higher energy demand due to their higher growth rate compared to their wild ancestor JF (93). However, its inconsistent regulation (increase in the JF and decrease in the broiler-based research lines) under heat stress condition is intriguing, particularly when heat stress reduced feed intake in modern broilers but not in JF (31). This suggest that central AMPK is probably not involved in feed intake regulation in chickens, since intracerebroventricular administration of 5-aminoimidazole-4-carboxamide-1-β-d-ribofuranosid (AICAR, AMP analog) or compound C (AMPK inhibitor) altered feed intake in chicken independently of AMPK activation (94).

Mechanistic target of rapamycin (mTOR) is also an evolutionary conserved and multi-tasking serine/threonine kinase that regulates various cellular process in response to growth factor stimulation, nutrient, energy or oxygen availability (95). The two best characterized downstream targets of mTOR are p70-S6 Kinase 1 (S6K1) and the eukaryotic initiation factor 4E (eIF4E) binding protein 1(4E-BP1). Of particular interest, although several genes of mTOR pathways were not affected in our study, hypothalamic S6K1 gene expression was higher in both modern broilers (95RB and MRB) under both environmental conditions. In combination with previous study in different species (rodents and Drosophila), our data suggest that central S6K1 may stimulate feed intake and protein synthesis in chickens in a HS-independent manner (23, 96).

As HSPs are molecular chaperones involved in regulating cellular homeostasis, stress response, and most recently feed intake *via* JAK2-STAT3 signaling pathways, we sought to determine here their hypothalamic expression profile. Surprisingly, our data showed that HS decreased the protein levels of HSP70 in all chicken lines, and HSP90 in JF, but it increased that of HSP60 in ACRB. Although further thorough studies are needed, these data indicate that central HSPs are probably regulated in a line-dependent manner in one hand, and the unexpected down regulation of HSP70 by heat load might be associated with adaptation and acclimatization of birds, on the other hand.

In summary, this is the first study to our knowledge showing modulation of hypothalamic expression of hypoxia (HIF complex)- and oxygen-associated molecules as well as their up- and down-stream mediators by heat stress in different genetically selected broiler-based research lines and their ancestor JF. The data indicate that hypoxia- and heat stress-responses are probably and tightly connected to feed intake regulation, which merit further in-depth investigations.

## Data Availability Statement

The original contributions presented in the study are included in the article/supplementary material, further inquiries can be directed to the corresponding author.

## Ethics Statement

The animal study was reviewed and approved by the University of Arkansas Animal Care and Use Committee under protocols 18083 and 16084.

## Author Contributions

SD conceived and designed the study. EG, TT, SO, and SD conducted the *in vivo* experiments. GB, AR, and CM performed the molecular and biochemical analyses. GB wrote the first draft of the manuscript. SD wrote the final paper with a critical review by all authors. All authors contributed to the article and approved the submitted version.

## Funding

This study was supported by a grant from the Arkansas Division of Agriculture, Animal Health Awards (to SD and SO), and from USDA-AFRI Sustainable Agriculture Systems (2019-69012-29905) to SD. The Arkansas Division of Agriculture and USDA-AFRI had no role in conducting the research, generating the data, interpreting the results, or writing the manuscript.

## Conflict of Interest

The authors declare that the research was conducted in the absence of any commercial or financial relationships that could be construed as a potential conflict of interest.

## Publisher's Note

All claims expressed in this article are solely those of the authors and do not necessarily represent those of their affiliated organizations, or those of the publisher, the editors and the reviewers. Any product that may be evaluated in this article, or claim that may be made by its manufacturer, is not guaranteed or endorsed by the publisher.
